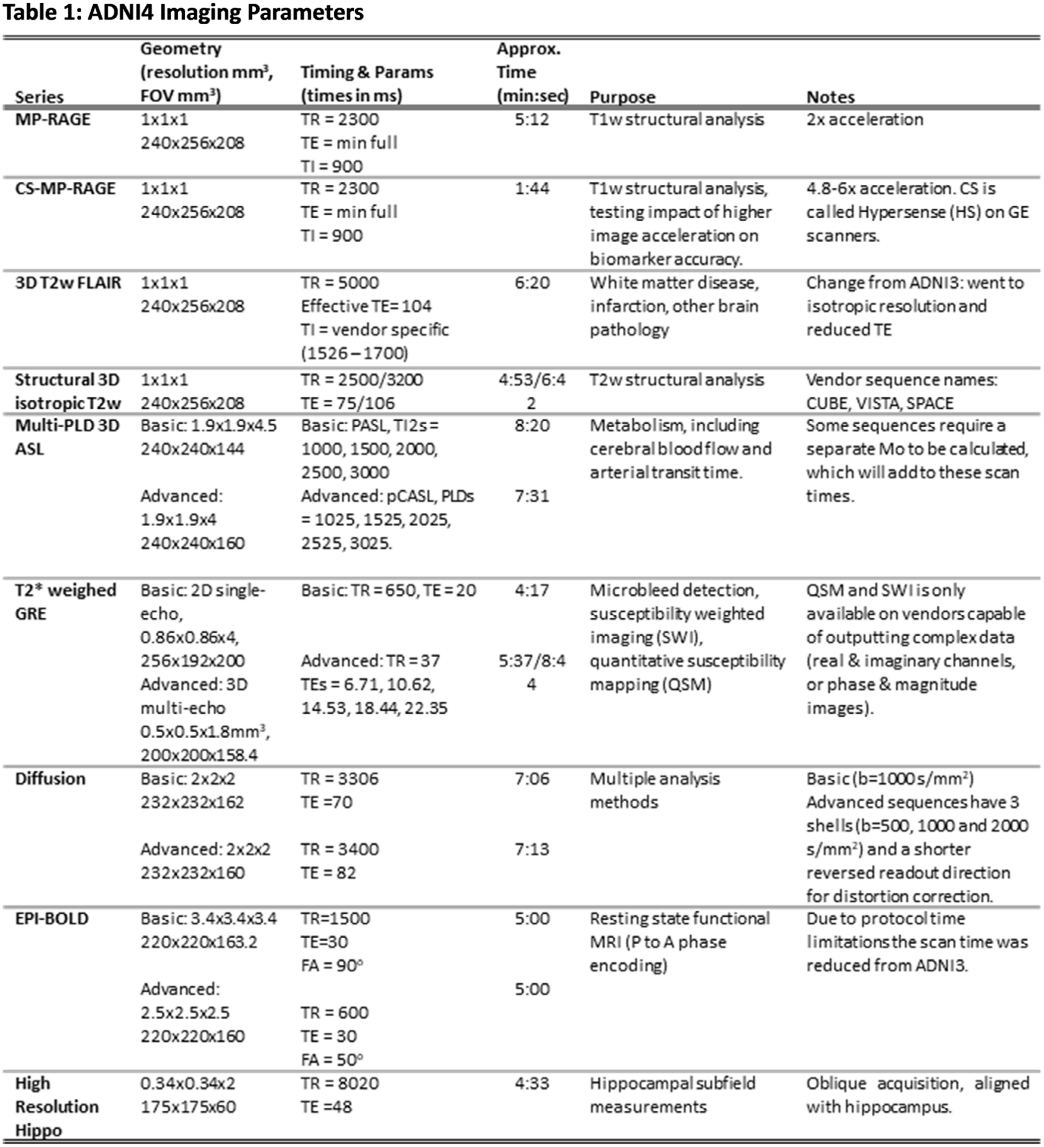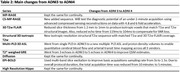# Design and Validation of the ADNI MR Protocol

**DOI:** 10.1002/alz.085261

**Published:** 2025-01-09

**Authors:** Arvin Arani, Bret J. Borowski, John Felmlee, Robert I. Reid, David L Thomas, Jeffrey L. Gunter, Randy L. Buckner, Youngkyoo Jung, Duygu Tosun, Michael S. W. Weiner, Clifford R. Jack

**Affiliations:** ^1^ Mayo Clinic, Rochester, MN USA; ^2^ Neuroradiological Academic Unit, Department of Brain Repair and Rehabilitation, UCL Queen Square Institute of Neurology, University College London, London UK; ^3^ Department of Radiology, Mayo Clinic, Rochester, MN USA; ^4^ Harvard University, Cambridge, MA USA; ^5^ University of California, Davis, CA USA; ^6^ Department of Veterans Affairs Medical Center, Northern California Institute for Research and Education (NCIRE), San Francisco, CA USA; ^7^ University of California, San Francisco, San Francisco, CA USA

## Abstract

**Background:**

Phase four of the Alzheimer’s Disease Neuroimaging Initiative (ADNI4) began in 2023. This time‐period corresponded to MRI vendors introducing product sequences with compressed sensing (CS), cross‐vendor adoption of arterial spin‐labelling (ASL) and multi‐band slice excitation, and hardware improvements (head‐coils, increased gradient amplitudes). These advances enabled the acquisition of new imaging measures and reduced scan times. The ADNI4 MRI protocol aims to maintain longitudinal consistency across two decades of data acquisition, while adopting new technologies. Here we describe the design and justification behind the ADNI4 protocol and list the target imaging biomarkers.

**Methods:**

The ADNI4 MRI protocol includes 9 MRI sequences; i) T1‐weighted (T1w) MP‐RAGE, ii) a compressed sensing T1w MP‐RAGE, iii) 3D T2w FLAIR, iv) structural isotropic 3D T2w v) T2*‐weighted susceptibility imaging vi) multi‐post labelling delay (PLD) 3D ASL, vii) 2D multi‐band‐multiple‐shell diffusion (dMRI), viii) 2D multi‐band EPI BOLD ix) high resolution, limited field of view, imaging centered on the hippocampus. Table 1 gives a detailed summary of the key protocol parameters and lists some of the imaging biomarkers that can be obtained from each series.

**Results:**

Structural imaging was kept consistent across imaging sites. Variations in vendor specific sequences, software upgrades, and hardware performance capabilities, resulted in variations in advanced imaging strategies. Table 2 highlights the advanced sequences introduced in ADNI4, which will gradually be implemented as the required upgrades and licenses are obtained at each site. The main advanced sequences that are new in ADNI4 from ADNI 3 are i) CS for T1w images, ii) pseudo‐continuous‐ASL (pCASL) being implemented on all 3 vendors (GE, Siemens, Philips), iii) multi‐PLD ASL, which allows for the generation of quantitative maps of cerebral blood‐flow (CBF) and arterial transit times (ATTs), iv) and isotropic 1mm^3^ resolution for 3D FLAIR. When ADNI4 began, product sequences that utilized artificial intelligence were not widely available and could not be adequately tested before the rollout of ADNI4.

**Conclusions:**

The ADNI4 database will continue to help the neuroimaging community, not only extract valuable imaging biomarkers, but provide the data necessary to test the impact of advanced imaging strategies on diagnostic accuracy and disease sensitivity.